# Molecular and Phenotypic Characterization of *Escherichia coli* Associated with Granulomatous Colitis of Boxer Dogs

**DOI:** 10.3390/antibiotics9090540

**Published:** 2020-08-25

**Authors:** Belgin Dogan, Shiying Zhang, Sarah E. Kalla, Esra I. Dogan, Cindy Guo, Chelston R. Ang, Kenneth W. Simpson

**Affiliations:** Department of Clinical Sciences, College of Veterinary Medicine, Cornell University, Ithaca, NY 14853, USA; be16@cornell.edu (B.D.); sz11@cornell.edu (S.Z.); sekalla@bcm.edu (S.E.K.); eid23@cornell.edu (E.I.D.); cg567@cornell.edu (C.G.); cra73@cornell.edu (C.R.A.)

**Keywords:** granulomatous colitis, Boxer dogs, *Escherichia coli*, adherent-invasive *E. coli* (AIEC), monocyte derived macrophages, fluoroquinolone, Crohn’s disease, CD48/SLAM, very early onset inflammatory bowel disease (IBD)

## Abstract

Invasive *Escherichia coli* is causally associated with granulomatous colitis (GC) of Boxer dogs and French Bulldogs. The virulence determinants of GC *E. coli* are unclear. *E. coli* isolated from 16 GC (36 strains) and 17 healthy control (HC: 33 strains) dogs were diverse in phylogeny, genotype, and serotype and lacked diarrheagenic genes. Genes encoding type II (*gsp*), IV (*traC*), and VI (*hcp*) secretion systems, long polar fimbriae (*lpfA*154/141), and iron acquisition (*fyuA*, *chuA*) were frequent in GC and HC. *E. coli* from 14/15 GC and 10/11 HC invaded Caco-2 better than non-pathogenic *E. coli* strain DH5α, with invasion correlated with motility and presence of *chuA* and *colV*. *E. coli* from all GC and 10/11 HC survived better than DH5α in J774 macrophages, with adherent-invasive *E. coli* (AIEC) in 60% GC and 73% HC. AIEC replicated in monocyte derived macrophages from a GC Boxer with CD48/SLAM risk haplotype but not the HC. Fluroquinolone resistant *E. coli* were less motile and invasive than fluoroquinolone sensitive (*p* < 0.05), and only 1/8 resistant strains met criteria for AIEC. In conclusion GC *E. coli* are diverse, resemble extraintestinal pathogenic *E. coli* (ExPEC), including AIEC, and can replicate in GC-susceptible macrophages. They are likely resident pathosymbionts that can opportunistically persist within macrophages of a GC-susceptible dog.

## 1. Introduction

Mucosally invasive *Escherichia coli* is causally associated with periodic acid-Schiff (PAS)-positive granulomatous colitis (GC) of Boxer dogs, French Bulldogs, and other breeds in the mastiff cluster [1,2,3]. Eradication of invasive *E. coli* with macrophage penetrating antimicrobials, such as fluoroquinolones and chloramphenicol, can induce clinical cures and long-term remission [1,4]. Conversely, failure to eradicate invasive *E. coli* is associated with a poor outcome [5]. The presence and regional distribution of granulomatous inflammation in the colon and ileum of dogs with *E. coli*-associated GC resembles Crohn’s disease (CD) [1,6,7]. However, the presence of PAS-positive macrophages in canine *E. coli*-associated GC also parallels intestinal inflammation in chronic granulomatous disease (CGD) and Whipple’s disease in people, which are similarly associated with persistent intracellular bacterial infections [8,9,10,11,12]. The predilection of GC for Boxers and French Bulldogs <3 years also mirrors CGD, which is considered a cause of very early onset inflammatory bowel disease (IBD) in children <6 years [1,13,14]. Impaired killing of catalase-producing bacteria in CGD is linked to mutations in nicotinamide adenine dinucleotide phosphate (NADPH) oxidase subunit genes [8,15,16], and persistence of *Tropheryma whipplei* is linked to an autosomal dominant mutation in interferon regulatory factor 4 (IRF4) [9]. Susceptibility of Boxers and French bulldogs to *E. coli*-associated GC is linked to a region encoding the CD48/SLAM family of genes on *Canis Familiaris* (CFA) chromosome 38, which is implicated in human IBD and the selective sensing and killing of *E. coli* by murine macrophages [3,17,18,19,20]. The bacterial attributes that enable GC *E. coli* to invade and persist within the colonic and ileal mucosa of a susceptible dog are unclear. Current knowledge is limited to four *E. coli* strains (CUKD1–4 isolated from two dogs) that lack genes associated with diarrheagenic *E. coli* [1,21]. Three of these strains (CUKD1–3) are similar in phylogeny (B2 and D) and genotype to extraintestinal pathogenic *E. coli* (ExPEC) [1,21] and can invade Caco-2 epithelial cells and persist in J774 macrophages resembling the adherent-invasive *E. coli* (AIEC) pathotype linked to CD in people and intestinal inflammation in murine models [1,22]. CD AIEC replicate more than non-pathogenic *E. coli* K12 in monocyte-derived macrophages (MDMs) from people with IBD (CD, ulcerative colitis (UC)) and healthy controls [23], and their persistence within MDMs is variably linked to polymorphisms in genes associated with CD and autophagy-related 16-like 1 (ATG16L1), immunity related GTPase M (IRGM), Unc-51 like autophagy activating kinase (ULK-1), and X-box binding protein 1 (XBP-1) [24,25,26,27]. The effect of the CD48/SLAM GC risk genotype on the ability of MDMs from Boxer dogs to kill AIEC is unknown. The emergence of GC *E. coli* that are resistant to multiple antibiotics, including fluroquinolones, correlates with persistent infection and poor clinical outcome [2,4,5], suggesting that fluoroquinolone resistant (FQ-R) GC *E. coli* may be more virulent than fluoroquinolone sensitive (FQ-S) GC *E. coli*.

The purpose of this study was to determine the relatedness, phylogeny, and virulence of GC-associated *E. coli* and their relationship to strains from healthy control (HC) dogs and pathogenic *E. coli* across species. Additionally, we explored the ability of GC-associated AIEC to persist in MDMs from GC and HC dogs, the effect of fluoroquinolone resistance on virulence, and the AIEC pathotype.

## 2. Results

### 2.1. GC E. coli are Diverse in Genotype, Phylogroup, and Serotype

We isolated 36 *E. coli* strains from GC dogs (1–4/dog/biopsy, median 2) and 33 from HC dogs (1–3/dog, median 2). *E. coli* from GC and HC dogs spanned the A, B1, B2, and D phylogenetic space (GC: 22% A, 36% B1, 17% B2, 25% D and HC: 6% A, 30% B1, 42% B2, 21% D). The prevalence of A, B1, and D was similar in GC and HC. Phylogroup B2 *E. coli* was more common in HC than GC (*p* < 0.05).

**Figure 1:**
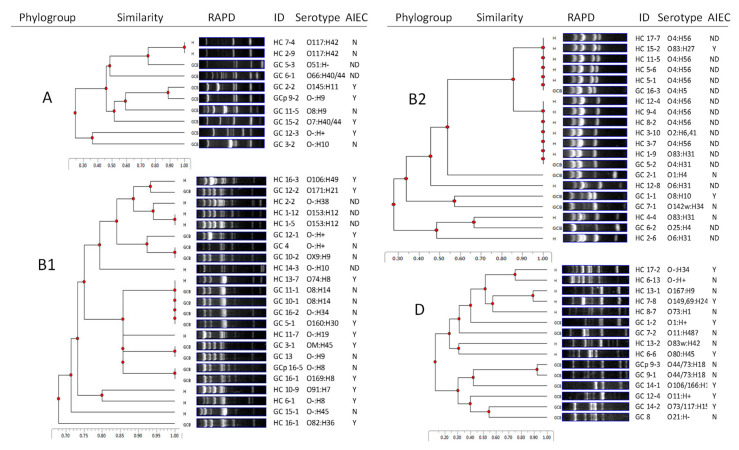
Relatedness of granulomatous colitis (GC) and healthy control (HC) *E. coli* according to the phylogroup and random amplified polymorphic DNA (RAPD) genotypes. Dendrograms were constructed for each phylogroup (A, B1, B2, and D) using TotalLab CLIQS software. Similarity values were generated for all pairwise comparisons. Serotype and adherent-invasive *E. coli* (AIEC) (Y/N) were indicated for each strain. Not determined (ND), since the *E. coli* strains were either cytotoxic or gentamicin resistant.

Genomic similarity within a phylogroup was evaluated by random amplified polymorphic DNA (RAPD)-PCR (Figure 1). Phylogroup A *E. coli* were isolated predominantly from GC, with only two isolates from HC (both were beagles), which shared an identical RAPD pattern on all three RAPD primers. Among phylogroups B1 and B2 *E. coli*, the same RAPD pattern (1283) was shared by several isolates that were further analyzed with two other RAPD primers, 1254 and 1290. Only two GC strains (GC 10–1 and GC 11–1) were found to be identical with the same serotype and virulence gene pattern. Similarly, four HC *E. coli* (HC 3–7, HC 8–2, HC 9–4, and HC 12–4) were genotypically identical, sharing one serotype and virulence gene pattern. None of the GC and HC *E. coli* shared a common RAPD pattern with RAPD primers 1254, 1283, and 1290. One GC dog was sampled before and after antibiotic treatment: *E. coli* strains GC 16–1 (pre-treatment) and GC 16–5 (post-treatment) were genotypically similar but had different serotypes.

Twenty different O-antigen serotypes were identified in 35 GC *E. coli* strains. Nine strains did not react with *E. coli* standard antisera and classified as O negative (O-ve). O-ve (*n* = 9) and O8 (*n* = 4) were the most common serotypes among GC *E. coli* (Figure 1). Additional serotypes were found in ≤2 strains. Sixteen different O-antigens were identified among 33 HC *E. coli* strains. The most common serotype was O4:H56 (*n* = 8), isolated from six HC dogs. Only three O-antigen serotypes were shared among GC and HC *E. coli* (O4, O73, and O106). Three HC *E. coli* were serotype O83, the serotype of prototypical AIEC strain LF82 (Figure 1) [28].

### 2.2. HC E. coli Cluster with UPEC

Multilocus sequence typing (MLST) analysis of *E. coli* from GC and HC with pathogen-associated serotypes O2, O4, O6, and O83 revealed a cluster of 8 HC *E. coli* with O4:H56 serotype associated with uropathogenic *Escherichia coli* (UPEC) (Table S1).

### 2.3. E. coli from GC and HC Resemble ExPEC and AIEC in Gene Content

We evaluated the prevalence of 27 virulence genes (including adhesins, toxins, iron acquisition, and secretion systems) by PCR. Diarrheagenic virulence genes were detected in only 1/36 (*eae*) and 1/33 (*stA* and *stB)* GC strains. The most prevalent virulence genes identified in GC were type II (*gsp*, 72% GC), type IV (*traC*, 56% GC), and type VI (*hcp*, 58% GC) secretion systems, long polar fimbriae *lpfA* (50% GC), iron acquisition genes *fyuA* (44% GC), and *chuA* (42% GC) (Table 1). There was no difference in the prevalence of these genes in *E. coli* isolated from GC vs. HC (Table 1). The prevalence of genes associated with the AIEC pathotype was also similar in GC and HC: *lpfA_141_* (17% GC, 15% HC), *lpfA_154_* (44% GC, 45% HC), *pduC* (19% GC, 36% HC), *ibeA* (3% GC, 9% HC), and *pmt1* (3% GC, 9% HC).

ExPEC-associated virulence genes *papC* (pap fimbriae), *sfaDE* (sfa fimbriae), and *cnf1* (cytotoxic necrotizing factor 1) were more prevalent in *E. coli* from HC (*p* < 0.05) than GC and were associated with B2 phylogroup, and O4 and O6 serotypes (*p* < 0.05). *ratA* and *traC* were also more prevalent in HC compared to GC (*p* < 0.05) (Table 1).

### 2.4. E. coli from GC and HC Invade Caco-2 Epithelial Cells and Survive in J774 Macrophages

**Figure 2:**
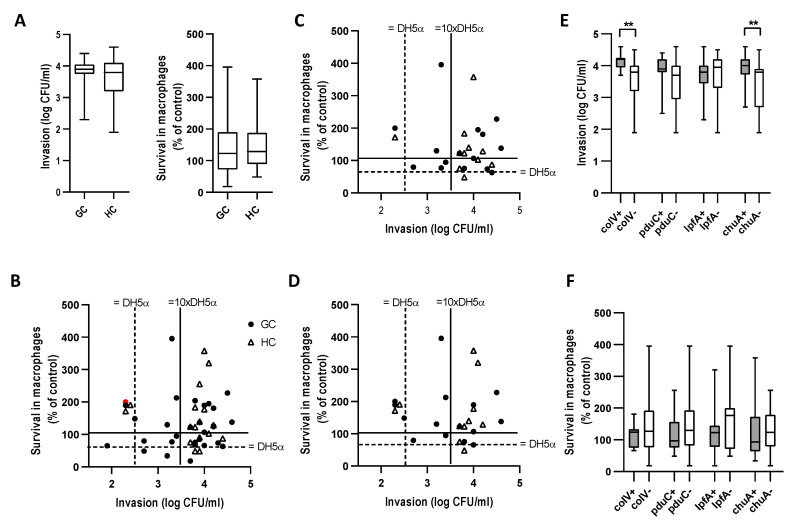
Ability of *E. coli* strains to invade Caco-2 colonic epithelial cells and survive in J774 macrophages. (**A**) Box plot of invasion and survival of all *E. coli* strains from GC (*n* = 30) and HC (*n* = 17) dogs. (**B**) Epithelial cell invasion vs. survival in macrophages of all *E. coli* strains. Red data point is non-motile strain GC4. Invasion and survival results with (**C**) best invading (**D**), best surviving *E. coli* from each dog. Influence of AIEC associated genes on (**E**) invasion of Caco-2 epithelial cells and (**F**) survival in J774 macrophages. Macrophage survival (24 h) is expressed as a percentage of bacteria, recovered 1 h post infection. In each box-and-whisker plot, the black line denotes the median value, the shaded box denotes the interquartile range (IQR (25th–75th percentile)), and the whiskers extend to the minimum and maximum. The Mann–Whitney U test was used to compare differences between the groups. ** *p* < 0.01.

After excluding gentamicin-resistant and cytotoxic strains, 30/36 GC *E. coli* (15/16 GC dogs) and 16/33 HC *E. coli* (11/17 HC dogs) were evaluated in cell culture. *E. coli* from GC and HC displayed similar levels of epithelial invasion and survival in macrophages (Figure 2A,B). 14/15 GC and 10/11 HC dogs had at least one *E. coli* strain that invaded better than non-pathogenic *E. coli* strain DH5α (Figure 2C), and 15/15 GC and 10/11 HC dogs had at least one *E. coli* strain that survived better than DH5α (Figure 2D). 9/11 HC and 11/15 GC dogs had at least one *E. coli* strain that could replicate (>100%) in macrophages (Figure 2D). There was no correlation between Caco-2 invasion and survival in macrophages (Figure 2B–D).

### 2.5. AIEC are Prevalent in GC and HC

*E. coli* strains that were more invasive than DH5α and could replicate in macrophages were classified as AIEC: 9/15 GC, and 8/11 HC dogs had at least one AIEC strain. The AIEC strains showed no consistent association with phylogroup or serotype and were not clonal (Figure 1). The virulence gene content was similar between AIEC and non-AIEC (Table 1).

We next examined the relationship of genes previously associated with AIEC [21]: *pduC, lpfA, chuA*, and *colV* to the in vitro phenotype. The presence of *chuA* and *colV* was associated with increased invasion of Caco-2 epithelial cells but had no effect on survival in macrophages (Figure 2E,F). *pduC* and *lpfA* had no effect on the in vitro phenotype.

### 2.6. E. coli Motility Correlates with Invasion of Caco-2 Epithelial Cells but Not Survival in J774 Macrophages

**Figure 3:**
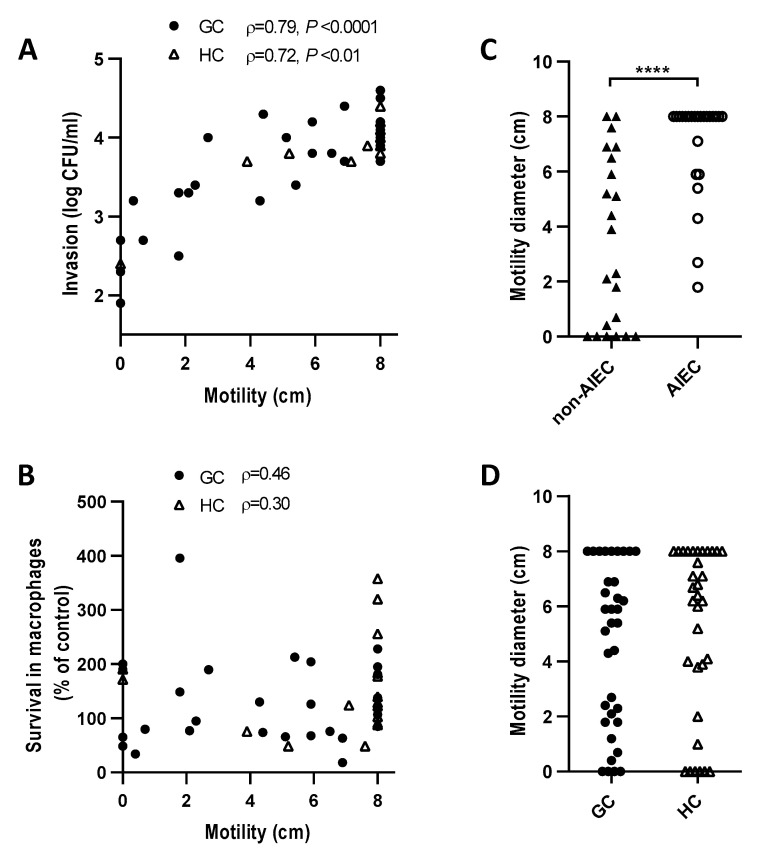
Relationship of motility to the invasion of epithelial cells and intracellular survival in macrophages. Motility versus (**A**) epithelial cell invasion, (**B**) survival in macrophages, (**C**) AIEC pathotype, and (**D**) disease. Motility was determined on soft agar. The correlation between Caco-2 invasion or survival in macrophages and motility was tested by the Spearman rank correlation. The Mann–Whitney U test was used to compare differences between the groups. Horizontal lines in C and D indicate the median values. **** *p* < 0.0001.

Motility has been linked to the invasion of human AIEC [29], prompting us to evaluate the relationship of motility to invasion of Caco-2 epithelial cells and persistence in J774 macrophages. We found a strong positive correlation between motility and epithelial cell invasion (Figure 3A) (*p* < 0001). Non-motile *E. coli* were about 500 times less invasive than the most motile *E. coli*. There was no correlation between *E. coli* motility and survival in macrophages (Figure 3B). Motility was also strongly correlated with the AIEC pathotype (*p* < 0.0001) (Figure 3C). There was no difference between the motility of *E. coli* from GC and HC *E. coli* (Figure 3D). Of note, one GC dog (GC4) with mucosally invasive *E. coli* was colonized by an *E. coli* strain that was non-motile and non-invasive but could replicate in macrophages (200%) (Figure 2B, red circle).

### 2.7. GC AIEC KD2 Can Replicate in MDMs from GC but Not HC

**Figure 4:**
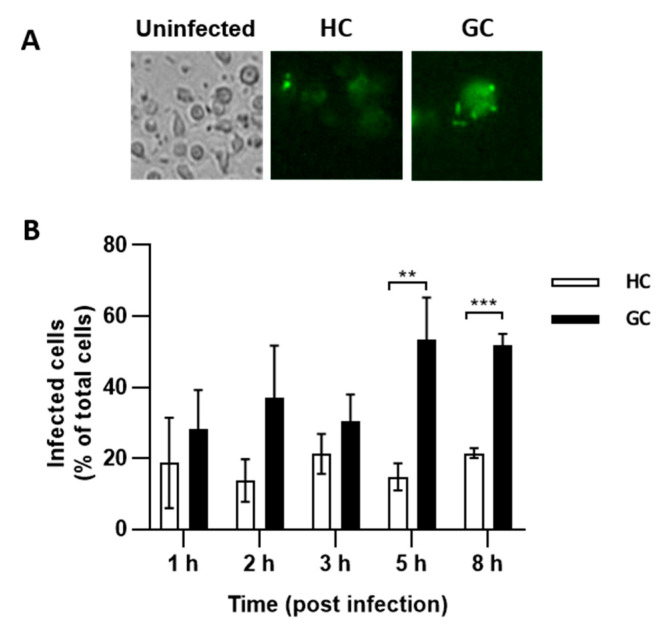
Killing of AIEC by monocyte derived macrophages (MDMs) from GC and HC. (**A**) Fluorescent microscopy images of GC and HC MDMs; uninfected and infected with a green fluorescent protein (GFP)-labelled AIEC strain CUKD2 at 5 h post-infection. (**B**) Number of infected MDMs as the percentage of total cells at various time points. ** *p* < 0.01, *** *p* < 0.001.

*E. coli* with an AIEC phenotype have been shown to persist and replicate in MDMs from people with CD, susceptibility linked to genetic polymorphism in autophagy (IRGM, ATG16L1, ULK-1, XBP-1) [23,24,25,26]. We infected MDMs isolated from a GC dog with the CD48/SLAM risk haplotype and a healthy Boxer >3 years with green fluorescent protein (GFP)-positive AIEC CUKD2. The number of MDMs containing GFP-positive AIEC CUKD-2 was higher (*p* < 0.05) in GC MDMs than HC MDMs at 5 and 8 h post-infection (Figure 4A,B). Extending quantification past 8 h was not possible due to death of MDMs. These data provide direct evidence of impaired killing of *E. coli* in a GC affected dog.

### 2.8. Fluoroquinolone Resistant E. coli (FQ-R) are Less Motile and Invasive than FQ-S

The high frequency of antimicrobial resistance in GC-associated *E. coli* suggests that FQ-R strains may be better adapted to invade and persist in the intestinal mucosa of a GC-susceptible dog. Antimicrobial sensitivity testing revealed that 15/16 and 7/16 GC dogs harbored at least one multidrug resistant (MDR) or FQ-R *E. coli* strain, respectively.

**Figure 5:**
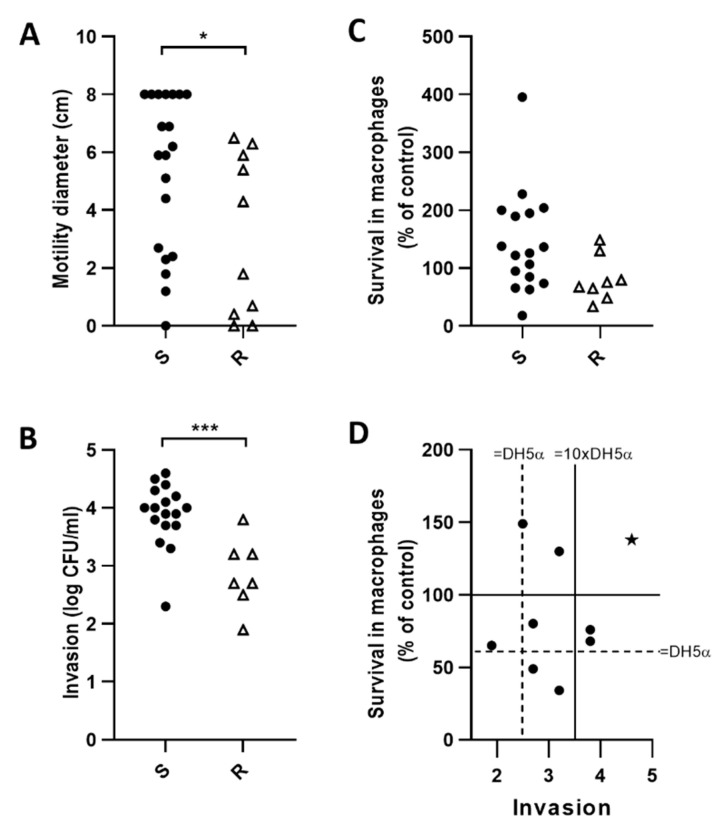
Effect of fluoroquinolone resistance on motility, invasion of epithelial cells, and survival in macrophages. (**A**) motility, (**B**) epithelial cell invasion, and (**C**) survival in macrophages. (**D**) Invasion vs. survival of FQ-R *E. coli* isolated from six dogs (seven biopsies; one dog was sampled before and after fluoroquinolone treatment). The star symbolled data point is fluoroquinolone sensitive (FQ-S) AIEC strain CUKD2. Mann–Whitney U, * *p* < 0.05, *** *p* < 0.001.

Fluoroquinolone resistant *E. coli* (11 strains from 7 GC dogs) were less motile and invasive than FQ-S (*p* < 0.05) (Figure 5A,B). There was no difference in survival in J774 macrophages (Figure 5C). The degree of invasion and survival of FQ-R *E. coli* (median, range) was 2.7, 1.9–3.8, 72%, and 34–149% respectively, with only 1/8 strains meeting criteria for AIEC (Figure 5D). As FQ-R strains were considered causal in six dogs that failed to respond to FQ (four were euthanized, two had persistent infection), our findings indicate that in vitro pathotyping of GC associated *E. coli* does not predict their ability to persist in the inflamed colon of a GC affected dog.

## 3. Discussion

The bacterial determinants that enable *E. coli* to invade and persist within the colonic mucosa of GC susceptible Boxer dogs and French Bulldogs are unclear. We sought to determine the phylogeny, relatedness, and virulence of GC associated *E. coli* and their relationships to strains from healthy dogs (HC) and the adherent-invasive *E. coli* (AIEC) pathotype. Additionally, we explored the ability of GC associated AIEC to persist in MDMs from GC and HC, the effect of fluoroquinolone resistance on virulence, and the AIEC pathotype.

We found that GC *E. coli* are diverse in genotype, phylogroup, and serotype, with no evidence of selection for a clonal virulent strain. *E. coli* from HC were also diverse and distributed across all phylogroups, though relatively enriched in B2 and UPEC-associated O4:H56 serotype strains, compared to GC. As the HC dogs in our study included a group of healthy beagles and hounds from the same environment, this may have amplified the association of the B2 O4:H56 cluster. However, the enrichment in B2, serotypes O2, O4, O6, and O83, and MLST associated with UPEC in HC echoes previous findings of *E. coli* from canine intestine/feces [30,31]. It is noteworthy that phylogroup B2, O83 serotype AIEC strains have also been repeatedly isolated from CD, e.g., prototypical AIEC LF82, NRG857c, and CU532-9 [28,32,33].

Analysis of virulence gene content revealed broad overlap in GC and HC *E. coli*, with genes associated with diarrheagenic *E. coli* notably absent. The most common virulence factors detected in GC were genes encoding types II, IV, and VI secretion systems (58–72% of strains) and variants of the adhesin *lpfA* (141/154) (50%). Secretion systems are used by various Gram-negative pathogens to transport virulence factors into the host and bacterial cells and secrete toxins and they play a role in epithelial cell adhesion, invasion, intracellular survival, and growth [34]. The LpfA adhesin is involved in translocation across Peyer’s patches and protective immune responses against *Salmonella* and is enriched in AIEC isolated from patients with CD [21,35,36]. Previous studies have variably linked *pduC*, *lpfA*, *pic*, *ibeA*, ColV, sideophores (*chuA*, *irp2*), *ratA*, *malX*, *kpsm*II, *papG*, *vat,* and *iss* to the AIEC pathotype [1,21,33,37,38]. We found no significant enrichment of virulence genes associated with the AIEC pathotype in GC vs. HC *E. coli*. Genes encoding UPEC associated adhesins, *sfaDE*, and *papG* were present in only 6% of GC *E. coli*, suggesting that they are distinct from UPEC. In contrast, HC *E. coli* were enriched in genes encoding UPEC associated adhesins *sfaDE* and *papG*, cytotoxic necrotizing factor *cnf1*, *traC* (type IV secretion system), and *ratA* (colonization of *Salmonella typhimurium)* relative to GC [34,39,40].

Previous analyses of *E. coli* strains from two GC affected Boxer dogs (CUKD1–4) revealed them as phylogroup A, B2, and D, with B2 and D isolates resembling AIEC associated with ileal Crohn’s disease [1,21]. In the absence of universal genetic markers, the AIEC pathotype is broadly defined by the absence of diarrheagenic virulence factors and toxins, and the ability to invade epithelial cells and persist in macrophages [21,22,33,41]. Using the criteria of invading Caco-2 epithelial cells better than DH5α and replicating in J774 macrophages [33], we found that the vast majority of *E. coli* isolated from the colonic mucosa of dogs with GC and HC were able to invade cultured epithelial cells or survive in macrophages better than commensal DH5α. *E. coli* strains with the ability to invade and persist (AIEC) were detected in 9/15 GC and 8/11 HC dogs. The presence of AIEC-like *E. coli* in GC and healthy dog intestine confirms our initial study of GC [1] and studies that have described canine enteric strains as being AIEC-like [31].

Analysis of virulence gene content of AIEC and non-AIEC isolated from GC and HC failed to yield genes associated with the AIEC pathotype, though we did find that *colV* and *chuA* were associated with increased invasion of Caco-2 epithelial cells. C*olV* has previously been associated with epithelial invasion by irritable bowel syndrome-associated *E. coli* [42]. We also found that motility of GC and HC *E. coli* correlated with the invasion of Caco-2 and the AIEC pathotype, in line with observations for CD associated AIEC [29,43]. In contrast we found no correlation between *E. coli* motility and enhanced survival in macrophages. Since the initial description of AIEC pathotype by Arlette Darfeuille Michaud [44], it has transpired that epithelial cell lines often used to define AIEC are HeLa contaminants (Intestine 407/HEp2) [45], and a wide variety of macrophage cell lines of human and murine origins have been used [41]. This lack of standardization of assays to determine the AIEC pathotype may explain the failure to reproducibly identify genetic traits associated with the AIEC pathotype [21,41,46].

While the AIEC pathotype is consistently associated with intestinal inflammation across species [21], the relevance of the in vitro cell culture-based characteristics used to define the AIEC pathotype to the pathogenesis of inflammation is unclear. For example, in ileal CD and canine GC, *E. coli* are not visualized within epithelial cells, questioning the importance of in vitro analysis of adhesion and invasion of epithelial cells [1,2,33]. As the hallmark of *E. coli*-associated PAS+ GC in dogs is the presence of *E. coli* within macrophages, and breed susceptibility is linked to genes in the CD48/SLAM region that selectively encode the sensing and killing of *E. coli* in mice [1,3,17,19,20,47]. The ability of GC-associated *E. coli* to persist in macrophages in vitro is likely disease-relevant. During the initial characterization of GC-associated *E. coli*, we observed that strains CUKD1–3 were unable to persist in murine bone marrow macrophages [1], but could replicate in J774 macrophages (derived from murine histiocytic sarcoma [48]), and fulfill the criteria for the AIEC pathotype [1]. While our results herein confirm that J774 cells are permissive to the intracellular replication of *E. coli* from GC and HC, this assay does not shed light on the ability of macrophages from GC-affected Boxers to kill *E. coli*. Our observation, that MDMs from a Boxer dog with the GC-associated CD48/SLAM risk haplotype [3] are more permissive to the replication of GC AIEC CUKD2 than MDMs from a healthy Boxer, supports the concept of impaired killing of *E. coli* in canine GC. Given the phenotypical similarities between canine GC and human CGD, it is relevant to note that the initial genome-wide association study (GWAS) of affected Boxer dogs identified neutrophil cytosolic factor 2 (NCF2), which is associated with CGD, as a candidate [49]. However, this was excluded by subsequent genotyping [3] and a normal oxidative burst in neutrophils from two GC affected dogs (The Neutrophil Monitoring Lab, NCI at Frederick, MD). Recent reports of intracellular *E. coli* in Boxers with concurrent PAS+ GC and PAS+ granulomatous cystitis (malakoplakia) and nephritis [1,50,51,52] suggest the potential for systemic inability to kill *E. coli* in GC-affected Boxers. The relative rarity of *E. coli*-associated GC and the need for fresh MDMs greatly limited our ability to perform functional analyses in more dogs, and we are working to extend evaluation of interactions between MDMs and *E. coli* prospectively.

Our findings suggest that cross-correlating the ability of GC-associated *E. coli* to replicate in GC MDMs and J774 could facilitate in vitro screening in J774 to predict the ability of *E. coli* to persist in vivo, e.g., CUKD2 (Figure 5D). Unfortunately, as dogs with GC were colonized with 1–4 different *E. coli* strains (median 2) and current fluorescence in situ hybridization (FISH)-based methods do not enable strain tracking, it is difficult to infer a specific casual strain. However, by integrating the results of antimicrobial susceptibility testing and the outcome of treatment, we were able to identify the most likely causal isolates in GC dogs colonized by an FQ-R strain, i.e., the presence of an FQ-R isolate in a GC dog that fails to respond to a fluroquinolone implies that the strain is associated with the disease [2,4,5,53]. Our finding, that FQ-R *E. coli* are less motile and invasive than FQ-S, which is similar in virulence gene content and J774 survival, with only 1/8 FQ-R categorized as AIEC, was surprising as we had speculated that the rapid increase in FQ-R GC *E. coli* may be a consequence of their enhanced ability to invade or persist in cultured cells. However, fluoroquinolone resistance has previously been linked to impaired biofilm formation in *Salmonella* and reduced ability of UPEC to cause cystitis and pyelonephritis [54,55]. This effect has been attributed to the impact of *gyrA* mutation, which is present in the majority of FQ-R GC *E. coli* [53], on the expression of virulence factors [55]. The ability of all but one FQ-R strain to persist better than DH5α but not replicate in J774 macrophages, despite being isolated from a patient with intracellular *E. coli*, suggested to us that the defect in *E. coli* killing, associated with GC and the CD48/SLAM risk haplotype, could be more permissive to replication than the J774 cell line. This possibility is supported by the results of experiments in CD48^−/−^ mice, where non-pathogenic human commensal *E. coli* F-18 persist and replicate, achieving levels 7-fold higher in CD48^−/−^ vs. wild-type (WT) macrophages [47,56]. This inability to contain non-pathogenic *E. coli* contrasts with reports of impaired killing of AIEC, but not non-pathogenic DH5α by MDMs from CD patients with CD-associated polymorphisms in autophagy genes that differentially favor the persistence (IRGM1, ATG16L1) and killing (XBP-1 and ULK-1) of intracellular AIEC and DH5α [23,26,27]. Taken as a whole, *E. coli*-associated PAS+ GC of young Boxer dogs and French bulldogs seems to more closely resemble very early onset IBD in people, which is commonly linked to monogenic primary immunodeficiencies, including those with impaired ability to kill bacteria that are normal residents, e.g., CGD, rather than later onset complex polygenic forms of IBD [13,14,26].

The route by which luminal *E. coli* gain access to mucosal macrophages in dogs with GC is unclear. In the intact intestine it could be related to an ability to invade epithelial cells, translocate between cells, or interact with Peyer’s patches, M cells, or dendritic cells. The presence of the adhesin LpfA in 50% of GC and 64% of FQ-R GC isolates suggests that Peyer’s patches and dendritic cells could provide a conduit to mucosal macrophages, which warrants investigation [21]. Our finding, that causally associated *E. coli* can be immotile and minimally invasive (GC4) and that FQ-R *E. coli* are less motile and invasive, questions the importance of epithelial invasion in the etiopathogenesis of GC [1,2,4,5]. Furthermore, in the context of an intact barrier, GC susceptible Boxer dogs that have recovered from GC still have *E. coli*, including FQ-R, in their fecal stream and colonic mucus [1,53]. The review of patient records reveals that GC affected Boxers frequently have a history of non-specific or parasite-associated bloody mucoid diarrhea in the first few months of life that fails to respond to symptomatic support [1,2,4,5,7]. At this time, we suspect that a non-specific inflammatory trigger, accompanied by *E. coli*-dominated luminal dysbiosis and loss of barrier integrity [1,57], leads to seeding of mucosal macrophages with impaired ability to kill *E. coli* and fuels an escalating cycle of inflammation and dysbiosis.

We conclude that GC *E. coli* are diverse, resemble ExPEC, including AIEC, in genotype and phenotype, and can replicate in GC-susceptible MDMs. While many strains from GC and HC meet criteria for AIEC, the ability of *E. coli* to persist within the mucosa of a GC susceptible dog does not directly correlate with the in vitro AIEC pathotype. The broad similarity of GC and HC *E. coli* suggests that GC *E. coli* are resident pathosymbionts that can opportunistically persist within mucosal macrophages of a GC-susceptible dog. Familial PAS+ *E. coli* + CD48/SLAM risk haplotype GC closely resembles very early onset, primary immunodeficiency related IBD and may serve to inform understanding of the etiopathogenesis and treatment of microbially driven intestinal inflammation across species.

## 4. Materials and Methods

### 4.1. Animals and Isolation of Bacterial Strains

Thirty-six *E. coli* strains isolated from colonic biopsies of 16 Boxer dogs with PAS+ *E. coli* associated GC (determined by histopathology and the FISH analysis) [1] and 33 strains isolated from rectal mucosal swabs of 17 clinically healthy dogs were evaluated by genotype and phylogroup. The *E. coli* isolation protocol and clinical characteristics of 14/16 GC and 17 healthy dogs (12 group housed beagles and hounds and 5 client-owned pets) have been reported previously [5]. *E. coli* from an additional two GC, one cultured before and one after antibiotic treatment, were included herein. Bacterial isolates were stored at −80 °C, and fresh non-passaged bacteria were used for all investigations. *E. coli* isolates were streaked on Luria–Bertani (LB) agar, and a single colony was inoculated into LB broth. Cells were grown overnight at 37 °C with shaking. All strains were tested for gentamicin resistance.

### 4.2. Molecular Characterization of E. coli

A total of 10–15 individual *E. coli* colonies were selected from each dog. Genotyping was performed by RAPD-PCR with primer 1283 [58], followed by primers 1254 and 1290 for strains with similar banding patterns [58]. Representative isolates that differed in overall genotype were selected for subsequent analyses. The major *E. coli* phylogenetic groups (A, B1, B2, and D) were determined by triplex PCR, as described previously [59]. *E. coli* isolates were serotyped for OH antigens and screened by PCR for the presence of genes encoding heat-labile toxin (LT); heat-stable toxins, STa and STb; shiga-like toxin types I and II, SLTI and SLTII; cytotoxic necrotizing factors 1 and 2 (*cnf1* and *cnf2*); and intimin-gamma (*eae*) at the *E. coli* Reference Center at Penn State University [60]. The presence of ExPEC and AIEC virulence genes *ratA*, *pmtI, colV*, *hcp, lpfA141, lpfA154, fyuA, aer (iucD), chuA, kpsMII, iss, malX, gsp, traC, afaBC, focG, ibeA, papC, sfaDE*, and *pduC* were determined by PCR, as described previously [21,61].

### 4.3. Multilocus Sequence Typing

Multilocus sequence typing (MLST) for seven loci (*aspC, clpX, fadD, icdA, lysP, mdh, uidA*) was performed on pathogen-associated O2, O4, O6, and O83 serotype strains, as described previously [62] and according to the established MLST protocols for *E. coli* (http://www.shigatox.net/ecmlst/protocols/index.html; EcMLST, Michigan State University). Sequences were submitted to the http://www.shigatox.net database for sequence type and clonal group identification.

### 4.4. Invasion and Persistence in Cultured Cells

Colonic epithelial cell line Caco-2 (ATCC HTB-37) and murine macrophage cell line J774 (ATCC TIB 67) were obtained from ATCC and grown according to ATCC protocols. The protocol for epithelial cell invasion and survival in macrophages has been previously published [33]. Gentamicin-resistant and cytotoxic strains were excluded from cell culture experiments. Gentamicin protection assay was performed to determine the invasive abilities of *E. coli* isolates. Briefly, Caco-2 cells were grown in 24-well plates for 7 days and confluent monolayers were infected with *E. coli* strains at a multiplicity of infection (MOI) of 20 for 3 h. Extracellular bacteria were killed with gentamicin (100 µg/mL) for 1 h and intracellular bacteria were enumerated, as described previously [33]. The invasion level was expressed as the total number of colony forming unit (CFU)/mL recovered per well. A non-invasive *E. coli* strain (DH5α) was used as a negative control.

Survival in J774 macrophages was determined after infecting cultured cells with *E. coli* at a MOI of 10 for 1 h. After the infection period, extracellular bacteria were killed with gentamicin at 100 µg/mL and gentamicin concentration was dropped to 20 µg/mL for longer infection periods. Survival after 24 h was expressed as the mean percentage of bacteria recovered after 1 h post-infection, defined as 100%. Gentamicin-resistant and cytotoxic strains or strains with known diarrheagenic virulence genes were excluded from cell culture assays.

*E. coli* strains were classified as AIEC if they invaded better than *E. coli* DH5α and survived and replicated in macrophages (i.e., ≥100%).

### 4.5. Ability of AIEC to Replicate in MDMs from GC and HC

The ability of GC-associated *E. coli* to persist and replicate in MDMs from GC and HC was determined using MDMs isolated from a PAS+, FISH + GC affected Boxer (F, age 1 year, 8 months) and a healthy control Boxer >3 years old. The GC-affected dog had been in remission following treatment with enrofloxacin.

MDMs were isolated from freshly collected whole blood (10 mL). The blood was gently layered on 10 mL of Histopaque-1077 (Sigma–Aldrich, St. Louis, MO, USA) in a 50 mL-Falcon tube and centrifuged for 30 min at room temperature at 400× g with no brake, following the manufacturer’s protocol. After centrifugation, the upper layer (plasma) was removed by pipetting. The second layer containing the buffy coat was transferred to 5 mL of PBS in a 10 mL centrifuge tube. After centrifugation at 250× g for 10 min, the cell pellet was washed again in 5 mL of PBS. Finally, the MDM cell pellet was resuspended in RPMI-1640 medium and plated in 24-well plate at a cell density of 5 × 10^5^ cells/well. The cells were incubated at 37 °C with 5% CO_2_.

For infection assays, we employed green fluorescent protein (GFP)-labeled AIEC CUKD2 (phylogroup D, serotype O1:H+, Genbank AJWP00000000) [1,21]. The GFP plasmid was constructed from the backbone of pZsGreen plasmid (Clontech, CA) with kanamycin as the antibiotic resistant gene. Competent *E. coli* CUKD2 was transformed with the GFP plasmid by electroporation, and the GFP-positive clones were selected on a kanamycin-containing LB plate.

Before infection, MDM cell media was removed and the cell surface was rinsed with warm PBS. An overnight culture of *E. coli* GFP-CUKD2 was diluted into fresh RPMI-1640 (Invitrogen) at a cell density of 1.5 × 10^6^ cells/mL (MOI of 30) and carefully added to MDMs at 1 mL/well. After 45 min incubation at 37 °C, the inoculum was removed and the cell surfaces were rinsed 2× with warm PBS. Fresh medium with 100 µg/mL gentamycin was added to the cells, followed by 60 min incubation of the cells at 37 °C. After 2× wash with PBS, the cells were given fresh medium with 20 µg/mL gentamycin and incubated at 37 °C for 24 h. During the infection, MDMs were monitored hourly and images were acquired at 1, 3, 5, and 8 h post-infection with a fluorescent microscope (Zoe, BioRad). Three to five images of each well at each time point were analyzed for the presence of GFP-positive bacteria. The number of cells containing GFP bacteria was determined for comparative analysis of GC and HC. GraphPad7.1 software was used for statistical analysis.

### 4.6. Motility

*E. coli* motility was evaluated on soft agar. *E. coli* was grown overnight at 37 °C in LB broth. A total of 2 μL of overnight culture was inoculated onto soft agar (1% tryptone, 0.5% NaCl, 0.25% agar) and plates were incubated at 30 °C overnight. Motility was assessed by measuring the diameter of the swarming area formed by each strain.

### 4.7. Antimicrobial Susceptibility Testing

Antimicrobial susceptibility of *E. coli* from 14/16 GC dogs has been reported previously [5]. Antimicrobial susceptibility of *E. coli* from one additional GC dog was determined as described [5].

### 4.8. Fluorescence in Situ Hybridization (FISH)

FISH analysis was performed, as described previously [1]. Slides were examined on a BX51 (Olympus America, Melville, NY, USA) epifluorescence microscope and images were captured with an Olympus DP-7 camera.

### 4.9. Statistical Analysis

Non-parametric tests were used to identify differences between groups. Mann–Whitney U or Fisher exact tests (2-tailed), when appropriate, were used. A value of *p* < 0.05 was considered statistically significant.

## Figures and Tables

**Table 1:** Prevalence of virulence genes in *E. coli* strains isolated from dogs with GC and HC.

Function	Genes	Disease		Pathotype ^#^		Fluoroquinolone Resistance		Phylogroup			
		GC (*n* = 36)	Healthy (*n* = 33)	AIEC (*n* = 24)	non-AIEC (*n* = 23)	S (*n* = 25)	R (*n* = 11)	A (*n* = 10)	B1 (*n* = 23)	B2 (*n* = 20)	D (*n* = 16)
Adhesins	*lpfA*_141_	17	15	25	13	24	0	0	26	25	0
	*lpfA*_154_	44	45	58	52	36	64	0	100	5	44
	*lpfA*	50	55	67	57	44	64	0	100	30	44
	*afaBC*	0	0	0	0	0	0	0	0	0	0
	*sfaDE*	6	33 *	0	0	8	0	0	0	65	0
	*papC*	6	33 *	0	0	8	0	0	0	65	0
	*focG*	3	15	0	0	4	0	0	0	30	0
Toxins	*sta*	0	3	0	0	0	0	0	4	0	0
	*stb*	0	3	0	0	0	0	0	4	0	0
	*stx1*	0	0	0	0	0	0	0	0	0	0
	*stx2*	0	0	0	0	0	0	0	0	0	0
	*cnf1*	6	36 *	0	0	8	0	0	4	65	0
Iron acquisition	*chuA*	42	64	42	43	44	36	0	0	100	100
	*fyuA*	44	48	29	30	36	64	30	13	95	44
	*aer*	17	3	7	19	8	36	20	4	5	19
Secretion systems (II, IV, VI)	*gsp*	72	64	63	78	72	82	40	70	55	75
	*traC*	56	91 *	63	74	48	73	70	70	75	75
	*hcp*	58	45	63	57	60	55	80	83	5	50
Various functions	*pduC*	19	36	33	43	20	18	10	30	30	31
	*kpsMII*	22	33	21	30	24	18	10	0	50	50
	*iss*	6	3	0	4	4	9	10	4	0	6
	*malX*	28	45	17	22	28	27	10	0	95	31
	*eae*	3	0	0	4	4	0	0	0	5	0
	*ibeA*	3	9	0	4	4	0	0	4	15	0
	*ratA*	22	55 *	21	26	16	36	10	0	85	50
	*pmt1*	3	9	4	9	4	0	0	9	5	6
	*colV*	22	45	21	17	28	9	10	0	90	25

Prevalence is shown as the % of strains that are PCR-positive for each gene. ^#^ Gentamicin-resistant and cytotoxic strains were excluded. Statistical differences in disease, pathotype, and fluoroquinolone resistance (FQ-R) categories were determined by Fisher’s exact test. * *p* < 0.05.

## Supplementary Materials

The following are available online at https://www.mdpi.com/2079-6382/9/9/540/s1. Table S1: Characteristics of *E. coli* strains with pathogen associated O2, O4, O6, and O83 serotypes.

## Abbreviations

Adherent-invasive *Escherichia coli* (AIEC); Crohn’s disease (CD); colony-forming unit (CFU); chronic granulomatous disease (CGD); extraintestinal pathogenic *Escherichia coli* (ExPEC); fluorescence in situ hybridization (FISH); fluoroquinolone resistant (FQ-R); fluoroquinolone sensitive (FQ-S); granulomatous colitis (GC); green fluorescent protein (GFP); genome-wide association study (GWAS); healthy control (HC); inflammatory bowel disease (IBD); Luria–Bertani (LB); long polar fimbriae (lpf); monocyte derived macrophages (MDMs); multidrug resistant (MDR); multilocus sequence typing (MLST); multiplicity of infection (MOI); O negative (O-ve); periodic acid-Schiff (PAS); phosphate buffered saline (PBS); random amplified polymorphic DNA (RAPD); ulcerative colitis (UC); uropathogenic *Escherichia coli* (UPEC), wild type (WT).
